# Mechanical Characterization and Production of Various Shapes Using Continuous Carbon Fiber-Reinforced Thermoset Resin-Based 3D Printing

**DOI:** 10.3390/polym16131828

**Published:** 2024-06-27

**Authors:** Md Zahirul Islam, Md Atikur Rahman, Luke Gibbon, Eric Hall, Chad A. Ulven, John J. La Scala

**Affiliations:** 1 Mechanical Engineering Department, College of Engineering, North Dakota State University (NDSU), Dept 2490, P.O. Box 6050, Fargo, ND 58108, USA; 2 Combat Capabilities Development Command Army Research Laboratory, FCDD-RLW-MD, Aberdeen, MD 57401, USA

**Keywords:** continuous carbon fiber reinforcement, light-assisted 3D printing, thermoset resin, print path optimization, complex shape printing

## Abstract

Continuous carbon fiber-reinforced (CCFR) thermoset composites have received significant attention due to their excellent mechanical and thermal properties. The implementation of 3D printing introduces cost-effectiveness and design flexibility into their manufacturing processes. The light-assisted 3D printing process shows promise for manufacturing CCFR composites using low-viscosity thermoset resin, which would otherwise be unprintable. Because of the lack of shape-retaining capability, 3D printing of various shapes is challenging with low-viscosity thermoset resin. This study demonstrated an overshoot-associated algorithm for 3D printing various shapes using low-viscosity thermoset resin and continuous carbon fiber. Additionally, 3D-printed unidirectional composites were mechanically characterized. The printed specimen exhibited tensile strength of 390 ± 22 MPa and an interlaminar strength of 38 ± 1.7 MPa, with a fiber volume fraction of 15.7 ± 0.43%. Void analysis revealed that the printed specimen contained 5.5% overall voids. Moreover, the analysis showed the presence of numerous irregular cylindrical-shaped intra-tow voids, which governed the tensile properties. However, the inter-tow voids were small and spherical-shaped, governing the interlaminar shear strength. Therefore, the printed specimens showed exceptional interlaminar shear strength, and the tensile strength had the potential to increase further by improving the impregnation of polymer resin within the fiber.

## 1. Introduction

In manufacturing industries, 3D printing has been experiencing significant growth as the creation of higher-performance materials has improved [1,2]. It has advantages over traditional manufacturing processes because of reductions in development time and costs. Additionally, it brings unparalleled design flexibility and the capability to manufacture complex geometries that are not possible to manufacture through conventional machining. Polymeric materials are the most commonly used materials for 3D printing.

Polymeric material used for 3D printing can be classified into two types: thermoplastics and thermosets. Thermoplastics (ABS, PLA, PA, and a few others) are most commonly used for fused-deposition-modeling (FDM) 3D printing because of their re-melting capabilities. In this printing process, semi-solid filaments are deposited in a layer-by-layer fashion to construct a specimen [3]. Due to the limitation of the constituent thermoplastic filament’s properties, FDM-printed items show poor mechanical strength. The tensile strength of FDM-3D-printed polymers is typically limited to a range of 20–45 MPa [4]. Hence, those FDM-printed items are primarily used as prototype products or toys.

Conversely, thermosets are commonly used as structural materials due to their better mechanical properties, chemical properties, and thermal stability [5]. Thermoset polymer materials undergo irreversible hardening when exposed to heating or UV irradiation. The most commonly used thermoset printing process is stereolithographic (SLA) printing, which employs vat photopolymerization. SLA printing processes utilize high-energy lasers to selectively cure photocurable thermoset resin in a layer-by-layer fashion and thus manufacture complex objects. Typically, acrylate-based thermoset resin with an appropriate photo-initiator is used for SLA 3D printing. Photo-initiators generate reactive species upon light exposure to initiate the free radical polymerization of resin [6]. The tensile strength of 3D-printed thermoset polymers is typically limited to a range of 25–70 MPa [7].

Furthermore, extrusion-based 3D printing of thermoset resin has also been demonstrated using a direct ink writing (DIW)-based 3D printing process. The DIW printing process does not require curing immediately after deposition, as feedstocks produce sufficient yield stress in order to maintain shape after deposition [8,9]. Print ink used for DIW-based printing requires proper rheological behavior. Therefore, this DIW-based printing process suffers from the limitation of nozzle clogging. However, UV-assisted extrusion-based DIW printing shows the potential to print with otherwise unprintable ink [10,11].

To further enhance the mechanical properties of 3D-printed thermoset plastics, different reinforcements (i.e., carbon black, filler, chopped fibers) have been mixed with thermoset resin to 3D print [12,13,14,15,16]. The tensile strength of short fiber-reinforced 3D-printed thermoset composites was reported to be below 100 MPa. Short fiber reinforcement increases the stiffness of the part, but the increase in strength is still limited as fiber pull-out occurs before fiber breakage [17]. Therefore, short fiber-reinforced 3D-printed composites still show inferior mechanical strength compared to conventional fiber-reinforced composites.

To achieve higher mechanical strength, continuous fiber was incorporated into extrusion-based 3D printing processes for both thermoplastic and thermoset matrix materials. The 3D printing of continuous fiber-reinforced thermoplastic composites has been well-established in the literature [17,18,19,20,21,22]. Matsuzaki et al. [23] 3D printed continuous carbon fiber-reinforced (CCFR) PLA composites using an in-nozzle impregnation process and demonstrated tensile strength of 185 MPa. Furthermore, Markforged commercialized 3D printing to fabricate continuous glass-, Kevlar-, and carbon fiber-reinforced thermoplastic composites utilizing pre-impregnated fibers [24,25]. Justo et al. [26] 3D printed CCFR thermoplastic (nylon) composites using a Markforged printer and obtained tensile strength of 700 MPa. However, due to the high melt viscosity of the thermoplastic matrix, continuous fiber-reinforced thermoplastics suffer from significant air void formation during printing [21,27,28,29].

Conversely, thermoset resins exhibit excellent wet-out of fibers during 3D printing with continuous fibers due to being a low-viscosity liquid at room temperature. Moreover, an in situ (in-nozzle) impregnation process of 3D printing of continuous fiber has been shown to have a good wetting ability for fibers [23]. CCFR thermoset polymers have wide applications in automobiles, aerospace, sports equipment, etc. due to their light weight, higher thermal stability, high specific strength, and modulus along the fiber direction [30,31]. In fact, fiber-reinforced thermosetting composites are used for high-performance applications because their low viscosity enables higher fiber volume fraction, and the cured nature of the resins reduces the tendency of creep under load relative to thermoplastic composites.

The 3D printing of CCFR thermoset composites was successfully demonstrated using direct ink writing (DIW)- and frontal propagation (FP)-based printing approaches [30,32,33,34,35,36,37]. Hao et al. [33] performed 3D printing of CCFR epoxy composites using a DIW printing approach and achieved tensile strength of 790 MPa. Additionally, Zhang et al. [35] 3D printed CCFR thermoset composites using a DIW printing process and reported tensile strength of 1140 MPa with a fiber volume fraction of 48%. To retain shapes after printing, the print ink for DIW and FP processes requires higher viscosity and, thus, a sufficient elastic modulus. Therefore, the preparation of resins for DIW and FP processes is critical and requires an optimal amount of rheology modifier. Some combinations of those formulations might create the issue of print nozzle clogging and require greater pumping efforts to pump those resins through the print nozzle [15]. Moreover, DIW-based printing has limitations in creating tall structures. As successive layers of ink are printed, the lower layers become unable to withstand the weight of the material above, leading to structural instability [38]. However, light-assisted 3D printing is less likely to encounter issues in printing tall structures due to its ability to instantly cure the resin while printing [11].

Light-assisted 3D printing enables the manufacturing of CCFR thermoset composites by utilizing low-viscosity, readily available thermoset resins. Abdullah et al. [39] demonstrated the 3D printing of CCFR composites using UV light assistance with an acrylate-based thermoset resin. Additionally, Rahman et al. [40,41] utilized UV light assistance in 3D printing CCFR thermoset composites and reported tensile strength of 230 MPa. To prevent resin curing and potential blockages at the nozzle tip, they focused UV light at a distance from the nozzle. However, due to the low viscosity of the resin, the uncured portion of the fiber retracted with the print nozzle. Therefore, the distance between the nozzle and UV light raised concerns regarding the printability of various shapes using the light-assisted printing process and low-viscosity resin.

The CCFR 3D printing of various shapes using engineered epoxy, which is solid at room temperature, was demonstrated in multiple research articles. This process involved melting the resin within the print nozzle, akin to in FDM [36,42,43,44]. Moreover, CCFR complex shapes were also 3D printed using a DIW-based printing approach. In this method, the printing ink had a higher viscosity, resulting in a higher elastic modulus, which aided in retaining shapes after extrusion onto the print bed [32]. However, printing complex shapes with low-viscosity thermoset resin remains challenging as the uncured portion of the fiber might retract with the print nozzle during the printing process.

The primary objective of this study was to design an overshoot-associated print path for a light-assisted 3D printing process, facilitating the fabrication of various shapes using low-viscosity thermoset resin. Additionally, this study conducted a mechanical characterization of the unidirectionally printed specimens to determine their tensile and interlaminar shear strengths. Moreover, void analysis of a printed specimen was performed to understand the size, shape, and distribution of voids present within the specimen and define their influence on tensile and interlaminar properties.

## 2. Experimental Methods

### 2.1. Materials

Teijin 3K continuous carbon fiber served as a reinforcement material for the 3D printing process. The carbon fiber was sourced from Teijin Carbon America, Inc. (Rockwood, TN, USA). Each individual fiber strand consisted of 3000 filaments, with each filament having a diameter of 7 µm. These fibers were characterized by a linear density of 200 tex (tex representing the weight in grams for 1000 m of fibers) and a density of 1.77 g/${cm}^{3}$. Furthermore, the carbon fiber boasted impressive mechanical properties, including tensile strength of 4100 MPa and tensile modulus of 340 GPa. Additionally, the elongation at break for these carbon fibers was 1.7%.

Commercially available photo-curable thermoset Peo-Poly Moai Tough Resin (urethane acrylate) was utilized as the 3D printing ink. This resin was procured from MatterHackers (Lake Forest, CA, USA) and could be photo-cured under light irradiation with a wavelength of 405 nm. The density of the resin was 1.15 g/${cm}^{3}$. To enable thermal curing along with photo-curing, 0.5% Luperox P was mixed into the resin before 3D printing. Luperox P (tert-butyl peroxybenzoate, 98%) was purchased from Sigma Aldrich (St. Louis, MO, USA). Both photo-curing and thermal-curing of the printing ink occurred through free radical polymerization reactions.

### 2.2. Rheology Test of Resin

The rheological characterization of the 3D printing ink was conducted using an ARES G2 rotational rheometer (TA Instruments, New Castle, DE, USA). The rheological test involved filling the resin between two 25 mm diameter parallel plate stainless steel fixtures with a gap of 0.5 mm between them. The test was carried out at the ambient lab temperature of 25 °C.

To determine the viscosity of the resin at different shear rates, a flow sweep test was performed. Additionally, a strain sweep test was conducted at a constant frequency of 6.28 rad/s to measure the storage and loss modulus of the printing ink. Before measuring any rheological parameter, the resin was allowed to equilibrate for 5 min.

### 2.3. Printing Process

A commercial gantry with moveable X-, Y-, and Z-axes was modified to create a custom 3D printer capable of producing CCFR thermoset composites. The printer utilized a stationary hot-rolled steel plate (300 × 300 mm) as its print bed. G-code, generated using the Python program, controlled the movement of the modified gantry. The print head of this customized printer included a specially designed print nozzle, a syringe pump (brand: LeTkingok, Shenzhen, China), and light lasers (0.8 W power, 405 nm wavelength; purchased from Sunshine Electronics, Dongguan, Guangdong, China).

**Figure 1:**
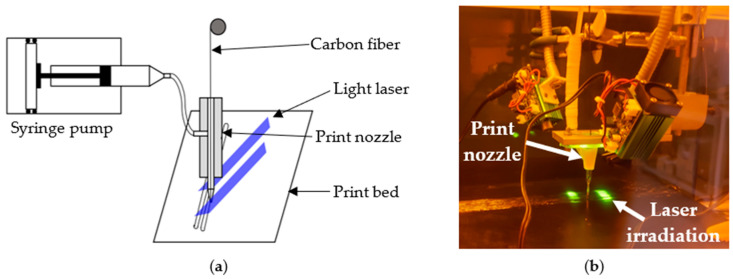
Light-assisted 3D printing process of CCFR thermoset composites, (**a**) schematic diagram of the printing process, and (**b**) real-life picture of the printing setup.

As depicted in Figure 1, a print nozzle was designed to simultaneously feed carbon fiber and thermoset resin onto the print head. Carbon fiber was pulled through the top of the nozzle, while thermoset resin was supplied from the side using a syringe pump. The resin flow rate (F) used during the printing process was 0.121 mL/min. A 14-gauge syringe needle with an inner diameter of 1.6 mm was attached to the tip of the nozzle to extrude fiber and resin together. A flexible needle tip was employed to ensure uninterrupted printing, even over irregularities.

Carbon fiber impregnated with resin was deposited on the print bed through the motion of the print head. To cure the resin on the print bed, an 800 mW 405 nm laser was employed. The laser was focused as a line at a specific distance from the print tip, as illustrated in Figure 1b. The laser’s focus distance was adjusted to prevent resin clogging at the nozzle tip. To ensure smooth resin flow during printing, a 0.85 mm gap was maintained between the nozzle tip and the print bed (or the previous layer). The gap between two consecutive lines of fiber on the same layer was set at 1 mm. The print head speed (S) during this printing process was 120 mm/min. Those print parameters were set by trial and error to ensure better print quality and superior mechanical performance. However, further optimization of these print parameters might have the potential to improve the print quality and mechanical performance even further.

A similar printing process was reported by the authors in previously published research articles [40,41]. Similar to the previous study, the same resin was utilized in this current research. However, this research employed 3K carbon fiber tows instead of the 1K carbon fiber tows used in the previous study. The incorporation of 3K carbon fiber tows eliminated the possibility of fiber breakage due to traction forces acting on the fiber during the printing process. Unlike the previous study, this research did not involve pre-impregnating the carbon fiber with the resin. Instead, raw carbon fiber was introduced into the print nozzle to be printed with liquid, photocurable thermoset resin.

Moreover, the resin flow rate and printing speed were adjusted to print composites with a higher volume fraction, reduced void content, and improved mechanical strength. The novelty of this research article lies in its elucidation of critical considerations for achieving the desired dimensional accuracy in printed objects, while also showcasing the overhanging capabilities of printed layers. Additionally, this study will illustrate algorithms designed for printing various shapes utilizing this particular printing process.

The amount of resin dragged along with the fiber during the printing process depends on both the resin flow rate (F, mL/min) and the printing speed (S, mm/min). However, using a single parameter to define the amount of resin dragged with the fiber during printing is more convenient. Therefore, in this current study, a parameter, $\frac{F}{S}$ (mL/mm), was defined, representing the amount of resin dragged per unit length of 3D printing. The parameter $\frac{F}{S}$ took into consideration the effects of both the resin flow rate (F) and the print speed (S). The value of $\frac{F}{S}$ used during the current printing process was 1.008 mL/m.

**Figure 2:**
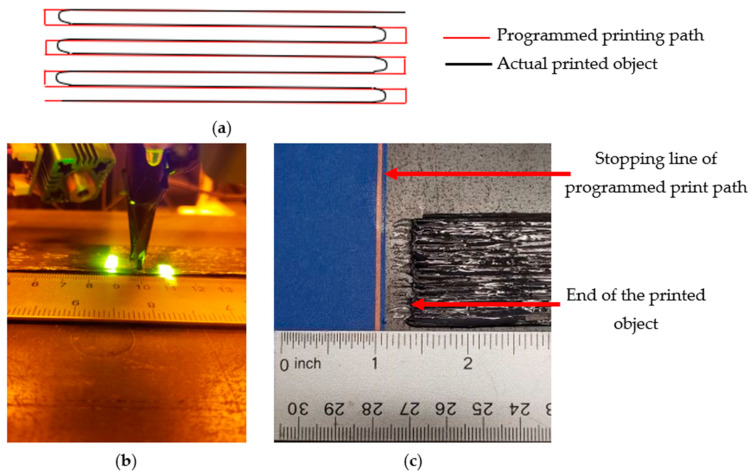
Dimensional accuracy analysis: (**a**) schematic comparison between programmed printing path and actual printed object; (**b**) distance between nozzle tip and laser irradiation; and (**c**) discrepancy between programmed printing path and actual printed object.

Figure 2a compares the nozzle’s programmed printing path generated by G-code with its actual printed trajectory. As depicted in Figure 2c, the 3D-printed object was slightly shorter than the intended programmed path. This deviation arose from the light sources being focused at a 10 mm distance from the print nozzle, as illustrated in Figure 2c. Consequently, the realized printed specimen measured approximately 10 mm (3/8 inch = 9.5 mm) less than the programmed path (illustrated in Figure 2c). In theory, the difference between the actual printed object and the programmed path should equal the nozzle-to-laser irradiation distance. However, the discrepancy was slightly reduced due to the fiber’s turning loop at the corners.

To achieve complete curing of the photo-irradiation-assisted 3D-printed CCFR composite, a thermal curing process was conducted in a convection oven at a temperature of 180 °C for 8 h. Photo irradiation partially solidified the resin, while thermal curing established a highly crosslinked polymer network [35,45].

### 2.4. Fiber Volume Fraction Measurement

The fiber volume fraction of the 3D-printed composites was measured by using carbonization of the matrix materials and an analytical approach. Carbonization of the matrix materials was performed using ASTM D3171 [46]. According to ASTM D3171 procedure H, the polymer matrix of the printed specimen was carbonized in a nitrogen environment, leaving fibers unaffected. Thus, this procedure enabled the calculation of fiber and matrix contents. Five specimens were tested. The average mass of each specimen was 0.9813 g. Crucibles with specimens were placed in a nitrogen-purging furnace (Model, RD4-KHE24, Warrington, PA, USA) at 565 °C for 5 h. However, some residual ash was left over after the carbonization of the matrix material. The carbonization ratio of the matrix material (${CR}_{m}$) was defined as the mass fraction of the ash left over after carbonizing the pure matrix material. TGA analysis showed that the ${CR}_{m}$ was 0.042 at the test temperature. Hence, the mass of matrix material ($m_{m}$) present within the specimen was calculated using Equation (1) and the mass of fiber ($m_{f}$) present within the specimen was calculated using Equation (2):

(1) $$m_{m}=\frac{m_{i}-m_{d}}{1-{CR}_{m}}$$

(2) $$m_{f}=m_{i}-m_{m}$$

where $m_{i}$ is the initial mass of the specimen before carbonization and $m_{d}$ is the final residue mass of the specimen after carbonization. Furthermore, the volume fraction of the printed composite was also evaluated by an analytical approach using the material properties. In the analytical approach, the mass of fiber ($m_{f}$) within a specimen was calculated using Equation (3):

(3) $$m_{f}=\frac{LWn\times tex}{10^{6}\times l}$$

where $m_{f}$ is the mass of fibers (g), L is the length of the specimen (mm), W is the width of the specimen (mm), n is the number of carbon fiber layers printed, tex is the fiber properties, and l is the gap between two consecutive fiber lines (hatch spacing, mm). Moreover, the mass of the matrix material ($m_{m}$) was calculated by subtracting fiber mass ($m_{f}$) from the total mass of the specimen (m).

Finally, in both approaches, the mass of the fiber ($m_{f}$) was divided by fiber density ($\rho_{f}$) to obtain the volume of the fiber ($v_{f}$). Afterward, the volume of matrix material ($v_{m}$) was calculated by dividing the matrix mass ($m_{m}$) by the matrix density ($\rho_{m}$). Hence, the overall fiber volume fraction ($V_{f}$) was calculated by taking the ratio of the fiber volume ($v_{f}$) to the total volume ($v_{f}+v_{m}$). Both approaches assumed zero void content for calculation.

### 2.5. Void Measurement

To obtain information about voids present within the printed specimen, a micro-CT scan was performed using a Phoenix X-ray scanner with a voltage of 80 kV. A seven-layer printed specimen was cut into a smaller section for micro-CT scanning. The dimensions of the specimen used for the micro-CT scan were approximately 12 mm in length, 12 mm in width, and 5 mm in thickness. The micro-CT scan was configured to detect voids with a minimum volume of 1.8 × $10^{-6}$ ${mm}^{3}$. The total volume of the material scanned was 696 ${mm}^{3}$. The result obtained from the micro-CT scan was analyzed using Volume Graphics (VG) software (version: MyVGL 2023.3.1).

### 2.6. Tensile Testing

To determine the tensile strength of 3D-printed composites, four-layer unidirectional specimens were printed using the printing process discussed in Section 2.3. The printed composites were cut into widths of 10 mm for tensile testing. Glass fiber tabs were attached at both ends of the specimen to ensure better load transfer and reduce stress concentrations at the grip. The tensile tests of the printed specimens were performed following ASTM standards (ASTM D3039 [47]). The test rate was 1 mm/min. A 25.4 mm extensometer was attached to the specimen to measure the failure strain of the specimen. Five specimens were tested under tensile load to calculate average tensile strength. The average thickness of the printed specimens was 3.2 mm.

### 2.7. Interlaminar Shear Testing

To measure the interlaminar shear strength (ILSS) of printed composites, seven-layer unidirectional specimens were 3D printed. Short beam shear (SBS) testing was performed according to ASTM D2344 [48] to measure the ILSS of the printed specimens. SBS testing is commonly performed to estimate the interlaminar failure resistance of fiber-reinforced composites [29]. Five specimens were tested to determine the average SBS strength. The thickness of the specimens was 5.1 mm.

A slightly thicker specimen consisting of seven layers was utilized for SBS testing to easily fulfill the dimensional requirements mentioned in the standard. As per the standard, the length and width used for the specimen were six and two times the thickness (30.6 mm and 10.2 mm, respectively), and the loading span used was four times the thickness (20.4 mm). The specimen was loaded in three-point bending, and the test rate used was 1 mm/min. The maximum load ($P_{m}$) during the test was calculated from the load–displacement curve. The short beam shear strength ($F^{sbs}$) was calculated using Equation (4), where b and h are the width and thickness of the specimen.

(4) $$F^{sbs}=0.75\times \frac{P_{m}}{b\times h}$$

## 3. Results and Discussion

### 3.1. Rheological Characterization

**Figure 3:**
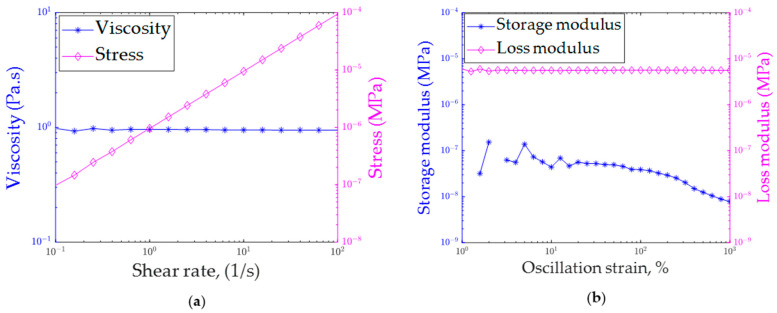
Rheological characterization of printing ink: (**a**) viscosity at different shear rates and (**b**) storage and loss moduli at different oscillation strains.

Figure 3 depicts the rheological characterization of the printing ink at various shear rates. As shown in Figure 3a, the viscosity of the printing ink remained constant regardless of the shear rate, with a value of 0.94 Pa∙s. Furthermore, the linear increase in shear stress with shear rate suggests that the printing ink behaved similarly to a Newtonian fluid. However, in DIW-based printing processes, the printing ink typically exhibits shear thinning behavior, where viscosity decreases as the shear rate increases. DIW-based printing inks typically exhibit viscosities of around 500 and 40 Pa∙s at shear rates of 10 and 100 (1/s), respectively [8].

Figure 3b illustrates the storage and loss modulus of the printing ink at various oscillation strains. The loss modulus was observed to have higher values compared to the storage modulus. A viscoelastic ink that has the capability to retain shapes after being printed usually exhibits a higher storage modulus than loss modulus. However, in the case of the printing ink used in this research, the loss modulus was higher than the storage modulus, indicating that the printing ink lacked this shape-retaining capability. However, the light-assisted 3D printing process had the potential to produce dimensionally accurate parts using low-viscosity thermoset resin that lacked a shape-retaining capability.

### 3.2. Volume Fraction

The average fiber volume fraction ($V_{f})$ of the printed specimen from the carbonization of the matrix material was calculated as 15.77%, with a standard deviation of 0.43%. Moreover, the average $V_{f}$ of the printed specimen using the analytical approach was 15.22 ± 0.11%. Both experimental and analytical measurements of $V_{f}$ exhibited very similar results. However, experimental analysis showed slightly higher values due to the lack of absolute fiber straightness in the printed specimen. The $V_{f}$ of the printed specimen could be further increased by optimizing print parameters such as hatch spacing, layer height, resin flow rate, and printing speed.

### 3.3. Overhanging Capabilities

**Figure 4:**
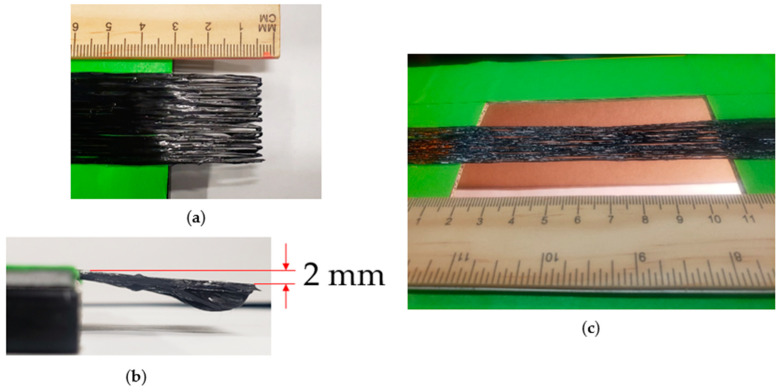
Overhanging capabilities of 3D-printed layers using light-assisted printing process: (**a**) cantilever overhanging (top view), (**b**) vertical deflection in cantilever overhanging shown in figure (**a**), and (**c**) overhanging between two supports.

Figure 4 demonstrates the overhanging capabilities of the printed carbon fiber layer. The resin-impregnated fiber tow hardened instantly upon laser exposure, thus having the capability of maintaining its shape even in mid-air. Figure 4a,b show the printed carbon fiber layer extending beyond the edge of the print bed by approximately 30 mm, with a vertical deflection of 2 mm. The printed layers beyond the edge of the print bed were able to retain their shapes. Supplementary Video S1 shows the printing of overhanging fiber lay-up. Furthermore, Figure 4c shows that the printed fiber layer formed a simply supported beam between supports located 100 mm apart. Based on the authors’ knowledge, this study is the first to demonstrate the overhanging capabilities of printed fiber layers. Such capabilities make it possible to 3D print various scalable complex structures, such as grid structures. Section 4 of this research study demonstrates the 3D printing of a grid structure utilizing the overhanging capabilities of the printed fiber tows. Moreover, these unique characteristics of the printed layer would be very helpful in creating complex structures while minimizing the need for support materials.

### 3.4. Surface Profile and Roughness

**Figure 5:**
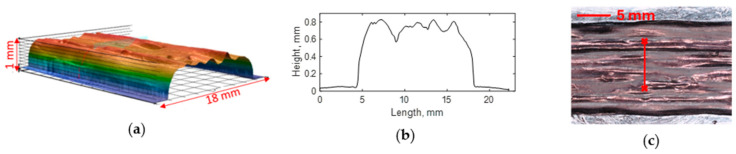
Surface profile measurement of a single 3D-printed layer: (**a**) 3D view of the printed layer; (**b**) 2D profile of the printed layer; and (**c**) line drawn on the printed layer to measure roughness.

To quantify the smoothness of the printed object, the surface profile and line roughness of a single 3D printed layer were measured using a Keyence digital microscope (Keyence, Model: VHX-7000, Osaka, Japan). Figure 5a shows the 3D surface view of a single printed layer and Figure 5b shows the 2D profile of the printed layer. The average layer height of a single layer was approximately 0.7 mm. Total height ($R_{t}$)—the vertical distance between the maximum profile peak height and the maximum profile valley depth—was 0.25 mm.

Figure 5c displays lines drawn on a printed layer to measure roughness, oriented transversely to the printing direction. The printed layer was coated with thin black pigment to avoid optical measurement errors due to transparent resin. Five measurements of $R_{a}$ were taken at the different locations on the printed layer. The calculated $R_{a}$ was 14.4 µm, with a standard deviation of 1.8 µm between measurements. The roughness of the FDM-3D-printed thermoplastic object varied between 15 and 60 µm [49]. Molten thermoplastic resin showed a higher melt flow index, while liquid thermoset resin had lower viscosity. Since lower viscosity thermoset resin spreads easily over surfaces, a better surface quality was achieved by utilizing liquid thermoset resin. Due to this superior surface roughness, these 3D-printed thermoset composites had the potential to meet surface roughness requirements as advanced structural elements. Based on the requirements, the surface quality of the printed CCFR thermoset composites could be further improved by depositing a layer of liquid resin on top of the carbon fiber layers.

### 3.5. Void Analysis

Micro-CT analysis indicated that the overall void volume fraction present within the printed specimen was 5.5%. Ye et al. [50] 3D printed CCFR thermoplastic composites and reported a void volume fraction of 12%. Moreover, conventionally manufactured composites contained a void volume of around 2 to 5% [51]. Therefore, 3D-printed CCFR thermoset composites exhibited a lower void content compared to their thermoplastic counterparts and were comparable to conventionally manufactured composites. Due to the enhanced flowability and wettability of liquid thermoset resin, CCFR thermoset composites exhibited a lower void content. However, knowledge of the overall void content is not sufficient to draw conclusions about their effect on performance; information about void size, shape, and location is also necessary [52].

**Figure 6:**
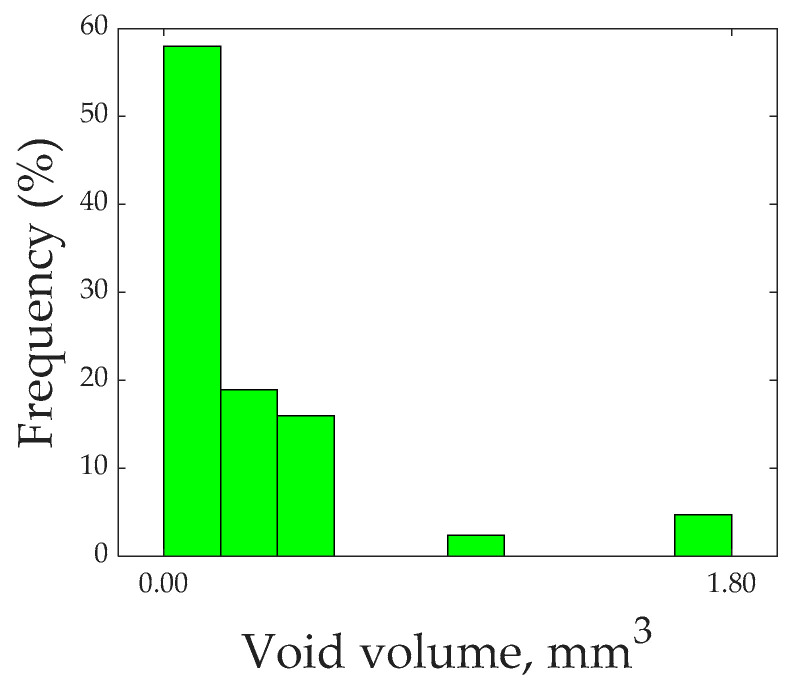
Histogram of void volume.

Figure 6 shows a histogram of the void volume. The overall range of void volume was classed into ten equal-sized groups, and the percentage of void volume present in each group is shown in Figure 6. The histogram makes it clear that 93% of the void volume comprised voids of individual sizes of less than 0.54 ${mm}^{3}$. Due to its lower viscosity, the printing ink flowed easily into all areas and showed less tendency to generate larger dry spots or voids.

**Figure 7:**
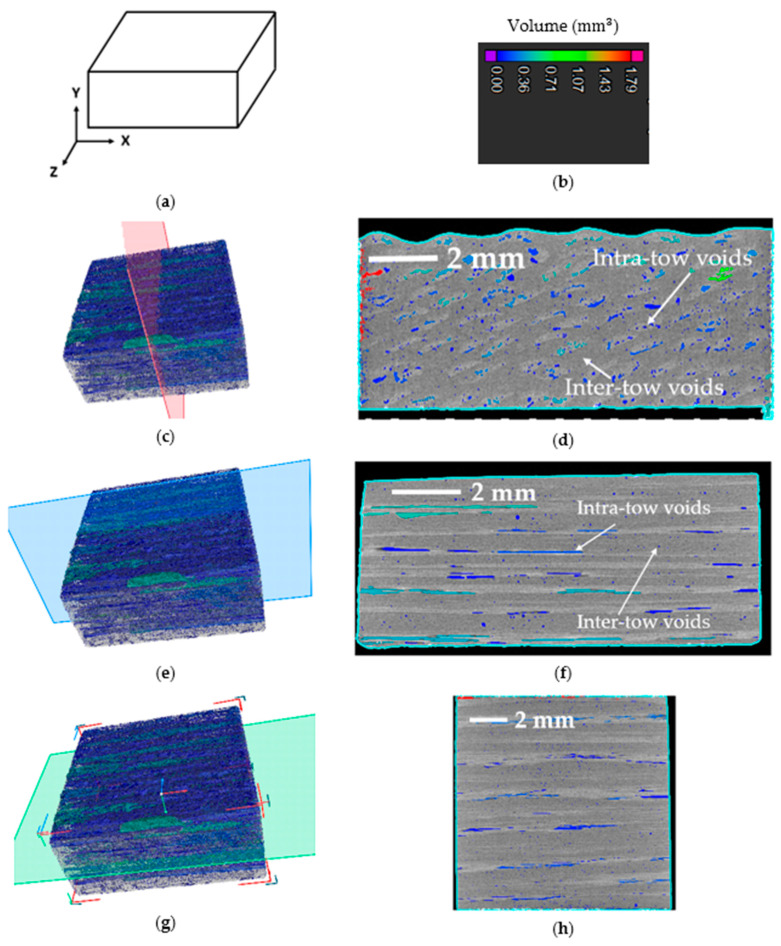
Sectional views of the specimen from micro-CT analysis, (**a**) specimen with co-ordinate axis, (**b**) color scale bar for void volumes, (**c**,**e**,**g**) sectional plane parallel to YZ, XY, and XZ plane, respectively, (**d**,**f**,**h**) sectional views corresponding to the subfigures (**c**), (**e**), and (**g**), respectively.

Figure 7 shows the sectional view of the specimen obtained from the micro-CT scan. Unidirectional fibers aligned along the X-axis, with Figure 7c showing the sectional plane parallel to the Y-Z plane, while Figure 7d shows the corresponding sectional views. Careful observation revealed that the majority of irregularly shaped voids were primarily located within the fiber tows, known as intra-tow voids, generated due to the poor impregnation of resin within the fibers [53]. Very few voids were observed between the fiber tows, known as inter-tow voids, generated due to the deformation and overlapping of fiber tows [53]. Moreover, thermal curing of the thermoset resin might have contributed to the formation of inter-tow voids.

Figure 7e shows sectional views parallel to the X-Y plane, while Figure 7f displays the corresponding sectional views. This section shows voids generated between the printed layers. It is evident from this section that the voids within the fiber tows were elongated and cylindrical in shape, whereas voids between the fiber tows were small and spherical. Figure 7g exhibits sectional views parallel to the X-Z plane, with Figure 7h presenting the corresponding sectional views. This section demonstrates voids generated between adjacent print lines. Cylindrical voids within the fiber tows and spherical voids between the fiber tows were also visible in this section.

Because of the low viscosity of the liquid printing ink, it flowed into all areas; therefore, inter-tow voids were lesser in volume and spherical in shape. Spherical voids were less conducive to crack propagation. Moreover, the fiber did not have sufficient opportunity to fully impregnate with the printing ink within the print nozzle. Hence, intra-tow voids were cylindrical in shape and larger in volume. Irregular or cylindrical voids predominantly contributed to crack propagation, leading to composite failure. Improving the impregnation of liquid resin within individual fiber filaments can help in reducing intra-tow voids. The impregnation of liquid resin within individual fiber filaments can potentially be enhanced by using several stretching rollers to pre-impregnate the fibers, as demonstrated by Ye et al. [54] for thermoplastic matrix materials and by Ming et al. [55] for thermoset matrix materials.

### 3.6. Tensile Strength

**Figure 8:**
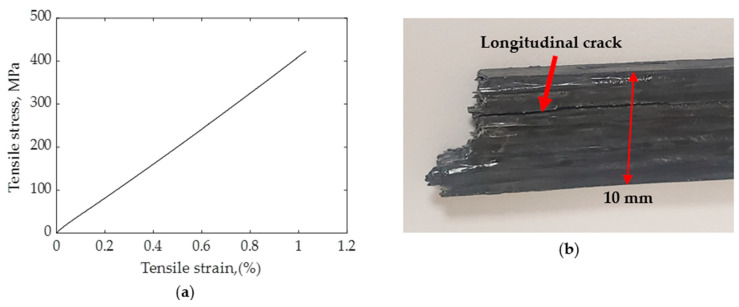
(**a**) Representative tensile test curve for continuous carbon fiber-reinforced 3D-printed thermoset composite and (**b**) fractured specimen.

The average tensile strength of the CCFR 3D-printed specimen was 390 MPa and the average tensile modulus was 42 GPa. The standard deviation for tensile strength was 22.3 MPa and the standard deviation for tensile modulus was 3.65 GPa. The failure stain ($\varepsilon_{c}$) of the specimen was 0.01 mm/mm (1%). The density of the printed composite was 1260 $kg/m^{3}$; therefore, the specific strength was 310 kNm/kg. In comparison, the specific strength of aluminum is around 220 kNm/kg. The superior specific strength of these CCFR 3D-printed composites makes them a promising material for advanced applications, including in aerospace and other industries. Figure 8a illustrates the representative tensile test plot of unidirectional CCFR thermoset composites, demonstrating linear stress–strain behavior until failure, which is typical for this type of composite material. Figure 8b shows a photograph of the failed specimen.

The theoretical tensile strength and modulus of the printed specimen were predicted using the rule of mixture (ROM) and the constituent properties provided in the manufacturer’s data sheet. Equations (5) and (6) present the formulas utilized for calculating theoretical strength:

(5) $$\sigma_{c}=\sigma_{f}^{\prime }V_{f}+\sigma_{m}(1-V_{f})$$

(6) $$E_{c}=E_{f}V_{f}+E_{m}(1-V_{f})$$

Here, $\sigma_{c\;}$ and $E_{c}$ represent the strength and modulus of the printed specimen. $V_{f}$ denotes the fiber volume fraction of the printed specimen (15.7%), $\sigma_{m}$ signifies the tensile strength of the matrix material (45 MPa), $E_{f}$ represents the modulus of the fiber ($E_{f}=$ 240 GPa), and $E_{m}$ indicates the modulus of the matrix material ($E_{m}$ = 1.5 GPa). $\sigma_{f}^{\prime }$ denotes the strength of the carbon fiber at the failure strain of the printed specimen.

According to the manufacturer’s data sheet, the strength of the continuous carbon fiber (Teijin, 3K) was 4100 MPa, with a failure strain of 1.7%. However, the failure strain obtained from the printed specimen was 1%, potentially due to the matrix cracking and the fraying of the fiber at the nozzle tip during printing. Matrix cracking caused failure before the fibers reached their failure strain. Evidence of matrix cracking was visible from the longitudinal crack formation during tensile failure, as shown in Figure 8b. Employing the unitary method, the strength of the carbon fiber corresponding to a 1% failure strain was calculated as $\sigma_{f}^{\prime }$ = 2412 MPa. Consequently, the calculated theoretical tensile strength and modulus of the printed specimen were 418.3 MPa and 39.1 GPa, respectively. However, the experimentally obtained tensile strength value was 6.7% lower than the corresponding theoretical values. This discrepancy was attributed to the inherent limitations associated with 3D-printed specimens, such as high void content (5.5%) and insufficient straightness of individual fibers within the composites.

Table 1 compares the tensile properties reported in this current study with those reported in the existing literature. To facilitate this comparison, the tensile properties cited in the literature were normalized for a volume fraction ($V_{f})$ of 16%. The normalization process involved utilizing linear regression, assuming zero strength at a zero percent volume fraction. The validity of employing linear normalization is supported by a study conducted by Zhang et al. [35]. In their study, they fabricated composites using 3D printing techniques at two distinct volume fractions (18% and 48%). The resulting tensile strengths of these printed composites (420 MPa and 1147 MPa, respectively) exhibited a predominantly linear relationship. Drawing on a study conducted by Genel et al. [56], conventionally manufactured carbon fiber–epoxy composites demonstrated strength and modulus values of 441 MPa and 42 GPa, respectively, at a fiber volume fraction of 16%. Conversely, Yang et al. [43] performed 3D printing of carbon fiber-reinforced epoxy composites using melted engineered epoxy. Their endeavor yielded strength and modulus values of 392 MPa and 28 GPa, respectively, for an equivalent volume fraction of 16%. Thus, the tensile properties reported in this current study are comparable with conventionally manufactured composites and composites 3D-printed using other technologies.

Additionally, Rahman et al. [41] demonstrated light-assisted 3D printing with continuous carbon fiber, exhibiting tensile strength of 232 MPa and a modulus of 22 GPa, with a fiber volume fraction of 13%. Moreover, Ming et al. [55] performed light-assisted 3D printing with continuous glass fiber and obtained tensile strength of 272 MPa and a modulus of 8 GPa. Therefore, this study revealed superior tensile strength (390 MPa) and modulus (42 GPa) values compared to the other light-assisted 3D-printed composites reported in the existing literature. However, Baur et al. [57] established a benchmark by performing light-assisted 3D printing of composites with tensile strength of 1599 MPa and modulus of 122 GPa, with a fiber volume fraction of 42%. By optimizing various print parameters such as hatch spacing, layer height, resin flow rate, nozzle diameter, and print speed, the currently reported printing process has the potential to increase fiber volume fraction and consequently enhance mechanical strength.

**Figure 9:**
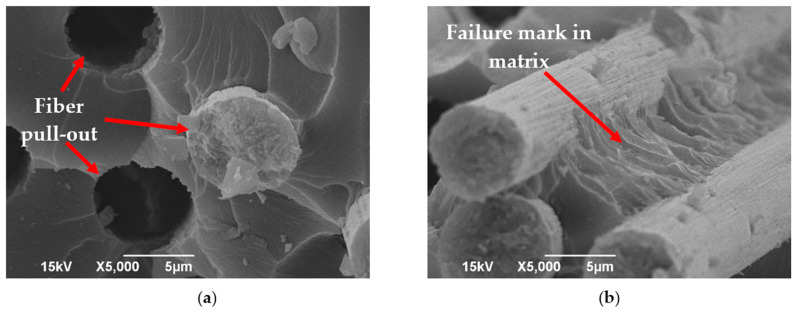
SEM images of fracture surfaces tested under tensile load: (**a**) evidence of fiber pull-out and (**b**) failure marks within matrix materials.

Figure 9 illustrates SEM images capturing the fractured surface of the specimen subjected to the tensile test. The principal mode of failure observed during tensile loading was fiber pull-out; however, predominantly isolated instances of single fiber pull-out were noted. Fiber pull-out happened due to the presence of numerous cylindrical-shaped voids present within the fiber tows. Single fiber pull-out occurred in the thermoset matrix, as noted by Zhang et al. [43], while thermoplastic matrix typically exhibited pull-out of large fiber bundles [43]. Furthermore, failure marks within the matrix materials pointed towards the gradual progression of failure through the matrix materials, which was attributed to the low number of spherical voids present between the fiber tows.

### 3.7. Interlaminar Shear Strength

**Figure 10:**
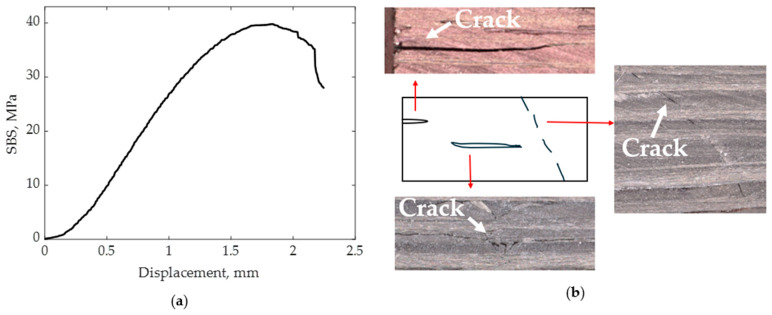
(**a**) Representative graph of the SBS test on the 3D-printed composites and (**b**) shear damage pattern of the specimens.

The interlaminar shear strength of the 3D-printed composites was evaluated by performing the short beam shear (SBS) test. The average SBS strength was found to be 38 MPa, with a standard deviation of 1.7 MPa between specimens. Figure 10a depicts the representative curve from SBS testing, showing the maximum strength at a flexure displacement of 1.8 mm, suggesting greater stiffness under shear loading for printed specimens.

The SBS strength of CCFR 3D-printed thermoplastic (nylon) composites was reported by Caminero [22], with a recorded strength of 32 MPa. Therefore, the CCFR thermoset composites 3D printed in this study exhibited an 18.8% higher SBS strength compared to their thermoplastic counterparts. Figure 10b shows the shear damage pattern of the printed specimens. The specimens underwent crack propagation through the matrix materials between fiber tows. SBS strength was primarily influenced by the matrix materials. The size and shape of voids in the matrix material greatly affected the SBS strength. As discussed in Section 3.5, the voids between fiber tows were spherical in shape and relatively small in size. Furthermore, spherical voids were less conducive to crack propagation. This accounted for the increased SBS strength observed in the printed specimens.

## 4. Custom Shape 3D Printing

**Figure 11:**
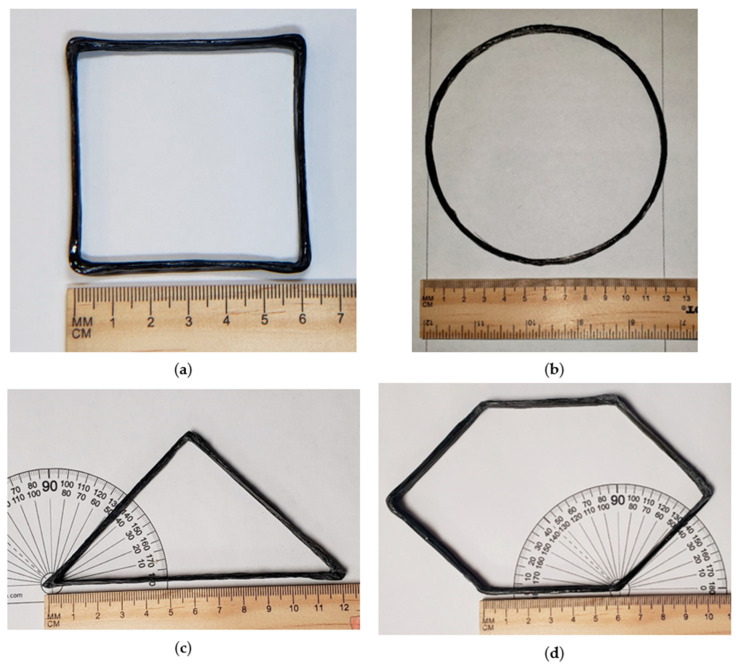
Custom shape manufacturing using light-assisted 3D printing of continuous carbon fiber with thermoset resin: (**a**) rectangular shape; (**b**) circular shape; (**c**) triangular shape; and (**d**) hexagonal shape.

Figure 11 shows the 3D printing of various shapes using the discussed 3D printing process of CCFR thermoset composites. Figure 11 shows 3D-printed square, circular, triangular, and hexagonal shapes. The shapes had 15 layers, with each layer having a height of 0.4 mm. The height of the printed shapes was 6 mm. Figure 11a shows the square shape; the expected/programmed edge length of the square shape was 60 mm. Actual edge length was measured using ImageJ software (https://imagej.net/ij/download.html, accessed on 1 June 2024). A virtual scale was defined in ImageJ software based on pixel numbers. The measured actual average edge length of the square shape was 59.8 mm. The expected diameter of the printed circular shape depicted in Figure 11b was 120 mm, and the actual measured diameter using ImageJ software was 116.8 mm. Figure 11c exhibits the printed triangular shape. The expected base length of the triangular shape was 120 mm with expected angles of 45°, 45°, and 90°. The measured actual base length of the triangular shape using ImageJ software was 118.5 mm, with a measured angle of 45 ± 0.5° at the base. Figure 11d shows the printed hexagonal shape. Each side of the hexagonal shape had an expected length of 60 mm, and the expected angles of the hexagon were 45° from the ground (X-axis). The measured actual edge length of the hexagonal shape using ImageJ software was within 60 ± 1.5 mm, with an angle of 45 ± 0.5° with a positive X-axis.

**Figure 12:**
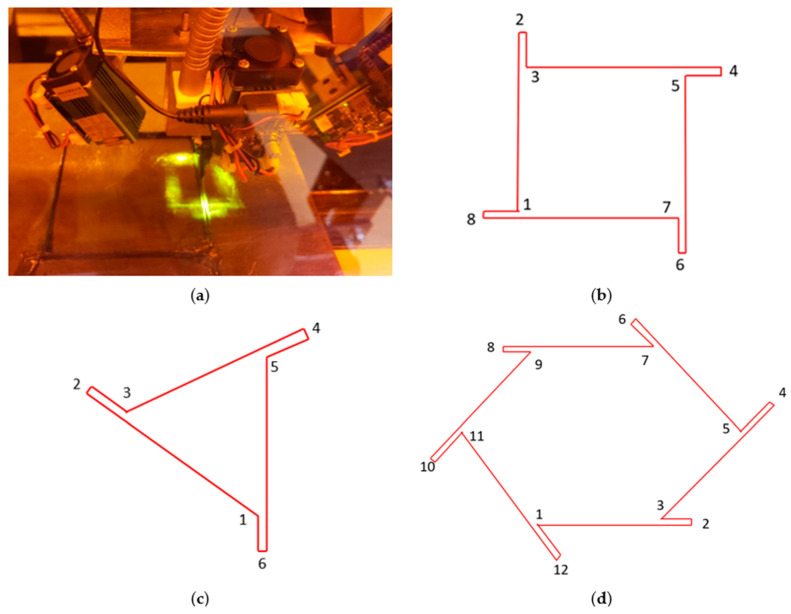
(**a**) Light–laser arrangement for printing irregular shapes, (**b**) programmed printing path for square shape, (**c**) programmed printing path for triangular shape, and (**d**) programmed printing path for hexagonal shape.

Figure 12a shows the position of the four-laser beam array focused on the four sides of the print nozzle. Laser beams were focused 10 mm away from the print nozzle. These focused laser beams formed a square around the nozzle tip.

Figure 12b shows the programmed printing path for the square shape. As the laser beam was placed 10 mm away from the printing nozzle, a section of print remained uncured, just adjacent to the print nozzle. Hence, at each corner, the print nozzle moved just past the edge of the object and then back to the original edge. The overshoot of the nozzle at each edge of the square shape is shown in Figure 12b.

As shown in Figure 12b, the programmed printing path was 1→2→3→4→5→6→7→8→1. The amount of overshot of the nozzle at each corner was equal to the distance between the print nozzle and the laser. Supplementary Video S2 shows the printing of rectangular shape with overshoot. A similar overshot was used while printing each shape.

Figure 12c shows the programmed printing path for the triangular shape with the overshoot of the print nozzle at each corner. The angles of the printed triangle were 45°, 45°, and 90°. The printing path was 1→2→3→4→5→6→1. The amount of overshoot on the angular edge (distance between points 2 and 3 and points 4 and 5) was 14.14 mm, which was equal to the diagonal distance between the nozzle tip and the laser irradiation. However, the overshot on the linear edge (distance between points 6 and 1) was 10 mm, which was equal to the horizontal/vertical distance from the nozzle tip to the laser irradiation.

Figure 12d exhibits the programmed printing path for the hexagonal shape. The overshoot occurred at each corner of the hexagon. The printing path was 1→2→3→4→5→6→7 →8 →9→10→11→12→1. Just as with the triangular shape, the overshoot on the angular edge was kept at 14.14 mm (distance between points 4 and 5, 6 and 7, 10 and 11, and 12 and 1), while the overshoot on the linear edge was kept at 10 mm (distance between points 2 and 3 and 8 and 9).

Such an overshot was not possible for the circular shape. However, the printed circular shape was able to maintain its shape with dimensional accuracy. The circular shape was programmed to have a 120 mm diameter, and the printed circular shape was able to retain similar dimensions, as shown in Figure 11b.

From the printed shapes in Figure 11, it is observable that corners were not perfect in terms of accuracy, as the fiber became a little misplaced or bent along the corners or turning points. This was because the focusing of the laser 10 mm away from the nozzle tip was not perfect since the focusing was manual. Moreover, the laser beam lines were defocused (spread over an area). Having a thin laser line beam with sufficient energy that could focus on an exact location would create an object with higher accuracy at its corners.

**Figure 13:**
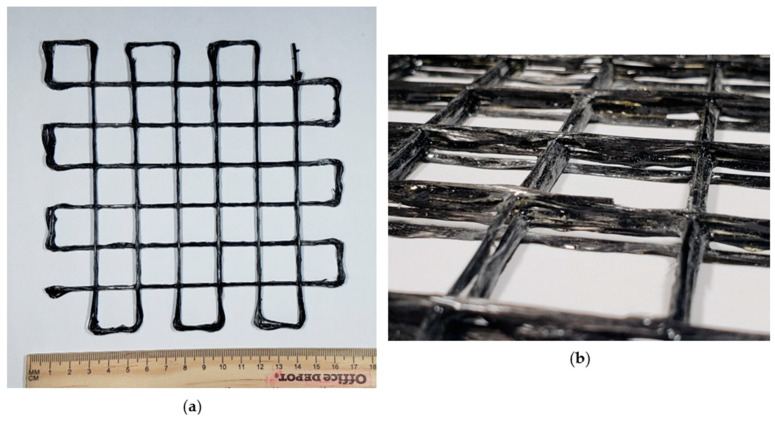
A 3D-printed grid pattern using continuous carbon fiber and thermoset resin: (**a**) grid structure and (**b**) exaggerated view.

Figure 13a demonstrates a grid structure printed using the discussed 3D printing process. Figure 13b illustrates an exaggerated view of the printed grid to visualize the edges more effectively. The overhanging capabilities of the continuous fiber during this printing process were also noticeable from this exaggerated view of the printed grid. The programmed/expected length of the side for each square box in the grid was 22 mm. The actual length of the side of each square box was measured using ImageJ software. Measurements were taken on 5 × 5 square boxes (total of 60 measurements). The average actual side length was 21.2 mm, with a standard deviation of 0.5 mm. The thickness of the overall grid structure was 5 mm.

**Figure 14:**
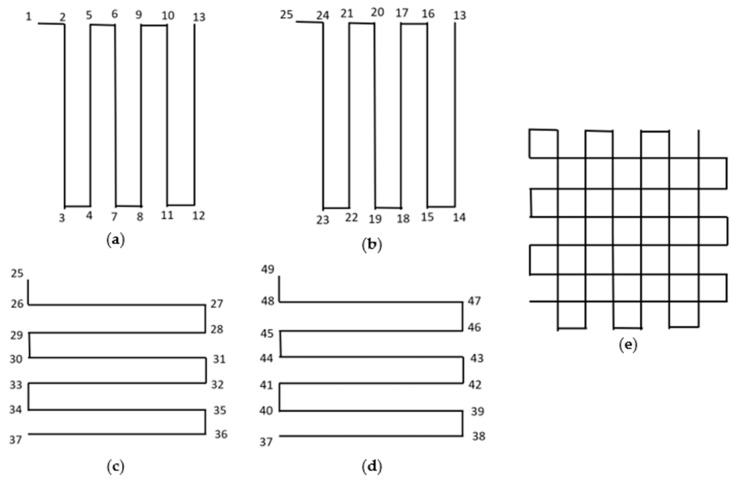
Schematic of printing path for grid structure: (**a**–**d**) actual printing path for one complete cycle and (**e**) printed structure after one cycle.

Figure 14a–d show schematics of actual printing paths for the grid structure and Figure 14e shows a schematic of the printed structure. After printing each path, the print nozzle moved up by a single layer height. Together with four printing paths (Figure 14a-d), it completed one single cycle of printing the grid structure. Each printing path overhung on the previously printed path. This overhanging capability made it possible to print a scalable grid following this algorithm. The demonstrated grid structure was created by completing three printing cycles. As discussed in the previous paragraph, the overshot in nozzle movement was required at each corner for printing the grid structure in order to compensate for the effect of distance between the print nozzle and the laser beam.

## 5. Conclusions

This study successfully demonstrated the 3D printing of various shapes using an overshoot-associated printing path with continuous carbon fiber and low-viscosity thermoset resin. Additionally, the ability to print fiber mid-air can enhance manufacturing processes by introducing customizable printing paths for products. The printed composite exhibited tensile strength of 390 ± 22 MPa and an interlaminar shear strength of 38 ± 1.7 MPa, with a fiber volume fraction of 15.7 ± 0.43%. Void analysis indicated that 5.5% of the overall volume was void within the printed specimen. Moreover, void analysis revealed the presence of numerous cylindrical-shaped voids within the fiber tows, potentially contributing to the reduced tensile strength of the printed composites. Therefore, improving the impregnation of resin within the fiber tows has the potential to yield 3D-printed composites with even greater tensile properties. The voids present in the matrix material between the fiber tows were relatively small in size and spherical in shape, which contributed to the development of exceptional interlaminar shear strength in the printed composites. Furthermore, the optimization of print parameters has the potential to increase fiber volume fractions and thus improve the mechanical properties of 3D-printed thermoset composites.

## Figures and Tables

**Table 1:** Comparison of tensile properties between CCFR 3D-printed composites in this study and those in the literature.

	Materials	Manufacturing Process	$\sigma_{c}$ (MPa)	$E_{c}$ (GPa)	$\varepsilon_{c}$ (%)	$V_{f}$ (%)	$\sigma_{c}$ at $V_{f}$ = 16%	$E_{c}$ at $V_{f}$ = 16%
Current study	Carbon fiber + acrylate	3D printing (light-cured)	390	42	1	16	390	42
Genel et al. [56]	Carbon fiber + epoxy	Conventional	826	78.7	1	30	441	42
Zhang et al. [35]	Carbon fiber + epoxy	3D printing (frontal propagation)	420	-	0.9	18	373	-
Yang et al. [43]	Carbon fiber + epoxy	3D printing (by melting engineered epoxy)	1372	98.2	0.85	56	392	28

## Data Availability

The original contributions presented in the study are included in the article/supplementary material, further inquiries can be directed to the corresponding author.

## Supplementary Materials

The following supporting information can be downloaded at: https://www.mdpi.com/article/10.3390/polym16131828/s1, Video S1: overhang printing, Video S2: rectangular shape printing with overshoot.
